# Supplementary material for: MALDI Mass Spectrometry Imaging—Prognostic Pathways and Metabolites for Renal Cell Carcinomas
Source: Cancers (Basel). 2022 Mar 30;14(7):1763. doi: 10.3390/cancers14071763 (PMC8996951; doi:10.3390/cancers14071763)
Supplement: Supplementary file 1 [file cancers-14-01763-s001.zip › cancers-1622671 supplementary material publish.pdf]

Table S1. Clinical and pathological characteristics of subtype chRCC.

Patient characteristics	***n* = 108**
Age median(range)(years)	65 (43-87)
Gender	
Male	47 (46.5%)
Female	54 (53.5%)
ISUP Grade	
Grade 1	NA
Grade 2	1
Grade 3	NA
Grade 4	NA
Pathological stage	
pT1	65 (65.7%)
pT2	25(25.3%)
pT3	9 (9.0%)
pT4	NA
pN+	2 (8.0%)
pM+	0
Survival (Dead/Alive)	16 (16%)/84 (84%)
Overall survival median (months)	38.5

Table S2. Clinical and pathological characteristics of subtype ccRCC.

Patient characteristics	***n* = 552**
Age median(range)(years)	65 (31-88)
Gender	
Male	195 (46.5%)
Female	285 (53.5%)
ISUP Grade	
Grade 1	16 (3.3%)
Grade 2	178 (36.5%)
Grade 3	149 (30.5%)
Grade 4	145 (29.7%)
Pathological stage	
pT1	277 (51.5%)
pT2	46(8.6%)
pT3	206 (38.3%)
pT4	9 (1.7%)
pN+	19 (15.4%)
pM+	4 (36.4%)
Survival (Dead/Alive)	171 (35.8%)/306 (64.2%)
Overall survival median (months)	36

Table S3. Clinical and pathological characteristics of subtype pRCC.

Patient characteristics	***n* = 122**
Age median(range)(years)	65 (29-75)
Gender	
Male	27 (26.7%)
Female	74 (73.3%)
ISUP Grade	
Grade 1	9 (7.4%)
Grade 2	62 (50.8%)
Grade 3	42 (34.4%)
Grade 4	9 (7.4%)
Pathological stage	
pT1	75 (67.6%)
pT2	19 (17.1%)
pT3	16 (14.4%)
pT4	1 (0.9%)

pN+	5 (20.0%)
pM+	NA
Survival (Dead/Alive)	27 (26.7%)/74 (73.3%)
Overall survival median (months)	36
